# A Case of a Metanephric Adenoma of the Kidney Surgically Treated with Robot-Assisted Laparoscopic Partial Nephrectomy

**DOI:** 10.1155/2013/703859

**Published:** 2013-09-19

**Authors:** Raunak D. Patel, Luke Frederick, Tobias Kohler, Bradley Schwartz

**Affiliations:** Division of Urology, Southern Illinois University School of Medicine, Springfield, IL 62702, USA

## Abstract

Metanephric adenomas are a rare neoplasm of the kidney with less than 200 cases reported. We report a case of a metanephric adenoma incidentally found on imaging in a 52-year-old Hispanic female and treated with robot-assisted laparoscopic partial nephrectomy. A brief review of the literature is also included.

## 1. Introduction

Metanephric adenomas of the kidney are a rare and most often benign neoplasm. They are most often removed surgically. The clinical presentation is similar to malignant renal masses and includes polycythemia, hematuria, abdominal pain, and a mass found incidentally on imaging. Less than 200 total cases have been reported to date, and though benign, an increase in the familiarity of this pathology may lead to less invasive treatments in the future.

## 2. Case Report

The patient is a 52-year-old Hispanic female that was referred to our clinic for an incidental left anterior midpole hypodense renal mass found on CT following a motor vehicle collision. The R.E.N.A.L nephrometry score of the mass was a 5a, with a size of 19 mm in the greatest dimension (Figure 3). The CT scan also showed two distinct left renal arteries, one inferior to the hilum and one at the superior aspect of the hilum near the mass itself. The renal vein was noted to coalesce distally. Her only complaint at the time of her visit to the clinic was intermittent left flank pain and anterior abdominal pain. She denied any history of gross hematuria or constitutional symptoms. Her past medical history was significant for hypertension and back pain and had no significant past surgical history. Physical examination revealed minimal left-sided abdominal tenderness and left-sided costovertebral angle tenderness. Significant preoperative lab findings included traces of RBCs and negative protein on urinalysis, and the hematology values were all within normal range except for slightly decreased hemoglobin of 35.7 percent.

**Figure 1:**
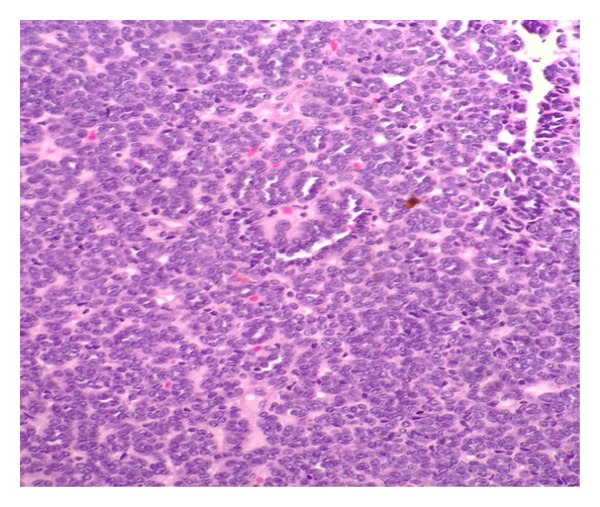
A micrograph of MA tissue at 200x magnification. There is a predominance of tightly packed small acini in the tumor.

**Figure 2:**
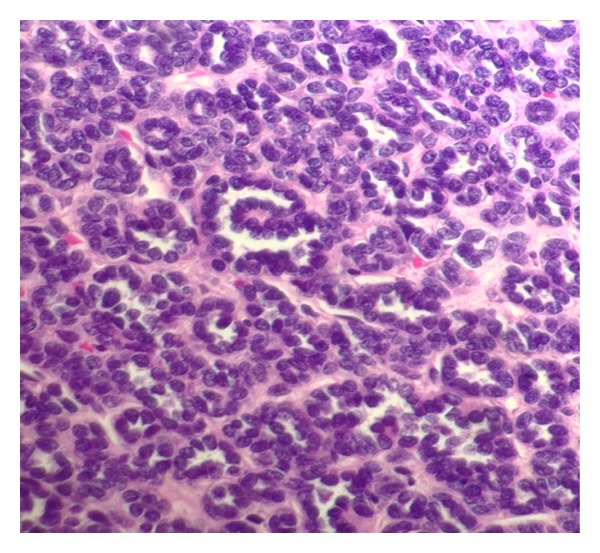
A micrograph at 400x magnification showing occasional glomeruloid structures in the center of the image.

**Figure 3:**
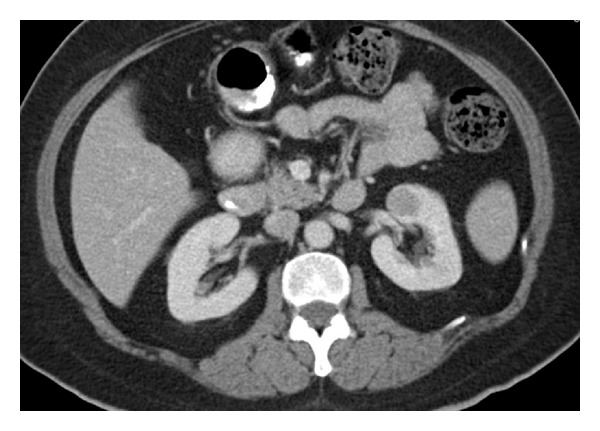
Contrast-enhanced CT showing a well-defined hypodense mass at the anterior midpole of the left kidney.

The patient was consented for a left robot-assisted laparoscopic partial nephrectomy. She underwent surgery without any acute complication or difficulty. The estimated blood loss was less than 100 mL, and the clamp time was thirty-eight minutes. She did well over her three-day hospital stay with no complications. Final pathology showed a benign metanephric adenoma (MA). Gross pathology demonstrated a 2.8 by 1.8 by 1.7 cm well-circumscribed, soft, and white-gray mass with a cut surface that was focally friable. The margins were negative. The mass did not penetrate through the renal capsule. The micrographs of our patient's tissue can be seen in the following figures, and were the primary source of our diagnosis. Findings included hyperchromatic cells, scant cytoplasm, tightly packed tubules, and glomeruloid-like structures (Figures 1 and 2). She was doing well when seen in the followup, and based on the current adult literature, a yearly renal ultrasound surveillance was planned.

## 3. Discussion

Metanephric adenomas are a rare type of renal epithelial neoplasm, closely related to other metanephric neoplasms including pure stromal lesions and metanephric adenofibromas [1]. Less than 200 cases have been reported worldwide [2]. The mean age of patients with MAs is 41 (5–83) [3]. MA is a benign, well-differentiated tumor in adults [1]. There has been one report in a 7-year-old child with an MA that had metastasis to the paraaortic, hilar, and aortic bifurcation lymph nodes [4]. Clinically, MAs are often incidental findings on imaging [5]. MA can present with hematuria, flank pain, or abdominal mass. Twelve percent of patients present with polycythemia vera which is higher than that of other renal neoplasms [2, 5]. Tissue cultured from a metanephric adenoma was found to produce significantly elevated concentrations of erythropoietin [6]. Though a benign process, it is important to quickly differentiate MA from other renal neoplasms as clinically they present in an identical fashion. MAs are treated as other renal masses with partial or radical nephrectomy.

There are no definitive radiologic findings in MAs that can differentiate them from other renal masses [7, 8]. It has been reported that there is a higher incidence of calcifications in MA (20%) than other renal neoplasms on CT, but this is not diagnostic [2]. Most often, they present as solitary well-circumscribed and well-defined tumors [2].

Histopathology of MAs has shown to reveal uniform small cells with scant cytoplasm, without mitosis, embryonic appearing, distributed in small round acini, and phenotypically similar to nephroblastomas [9]. One case of a 78-year-old was reported in which MA was diagnosed with a renal biopsy and was treated with surveillance. Micrographs showing rosette-like arrangements of small, blue cells with scant cytoplasm and evenly distributed, fine nuclear chromatin allowed for diagnosis of MA in this patient [10]. Four other studies showed similar histologic findings leading to the diagnosis of MA using fine-needle aspiration [3]. Renal biopsy can in this setting obviate the need for surgical intervention.

Historically, patients with metanephric adenomas treated with partial or total nephrectomy have an excellent prognosis. Due to its benign history and surgical treatment, the followup has been short and not well documented. However, one study suggested a similar followup of patients with MA as with those with renal cell Carcinoma (RCC) due to the finding of metastasis in the 7-year-old patient described previously [4]. Such surveillance includes clinical examination and a chest radiograph every six months as well as an abdominal CT scan after one year. Laboratory tests would be less useful in this setting.

## 4. Conclusion

In this case, we treated a benign MA with robot-assisted laparoscopic partial nephrectomy. MA cannot be differentiated from other malignant neoplasms based on imaging. Renal biopsy is an option in the appropriate setting. If no histologic diagnosis is available, MA should be treated as all other renal masses with partial or radical nephrectomy, cryoablation, or radiofrequency ablation. MA is easily recognized microscopically and differentiated from other renal neoplasms. Though a benign disease, followup is appropriate with radiographic imaging including chest radiograph and computed tomography.
